# Editorial: The Sustainability Challenge: New Perspectives on the Use of Microbial Approaches and Their Impact on Food and Feed

**DOI:** 10.3389/fnut.2020.00118

**Published:** 2020-08-25

**Authors:** Rossana Coda, Andrea Gianotti, Ana Gomes, Carlo G. Rizzello

**Affiliations:** ^1^Department of Food and Nutrition, HELSUS Institute of Sustainability Science, Helsinki, Finland; ^2^Department of Agricultural and Food Sciences - DISTAL, Interdepartmental Centre for Industrial Agri-Food Research, University of Bologna, Cesena, Italy; ^3^Universidade Católica Portuguesa, CBQF - Centro de Biotecnologia e Química Fina – Laboratório Associado, Escola Superior de Biotecnologia, Porto, Portugal; ^4^Department of Soil, Plant and Food Science, University of Bari Aldo Moro, Bari, Italy

**Keywords:** bioprocessing, fermentation, microbiome, sustainability, food, feed

Building a more resilient food chain, reducing food loss and waste, improving food production practices and increasing plant-based food consumption are some of the fundamental actions suggested in The Sustainable Development Goals adopted by the United Nations Member States in 2015^1^. The objective of this special issue was to explore how the use of microorganisms as direct or indirect sources of transformation could contribute to these sustainability practices. In this context, the following strategies have been presented: (i) valorization of side-streams and underutilized food resources via fermentation, (ii) improvement of the efficiency of bioprocesses for the food and feed industry, and (iii) understanding and applying the microbiome as a resource to improve the agro-food system.

Fermentation was used to upgrade the nutritional and functional value of cereal industry by-products and to promote less utilized but very valuable grains, such as pseudocereals. With a production above 2 billion tons per year, cereals provide a major food resource worldwide but also generate a great amount and variety of side-streams. Verni et al. provide an overview on lactic acid bacteria, yeasts, and fungi fermentation as biotechnological tools for processing cereal side-streams to improve their nutritional properties. Among the positive effects of microbial fermentation, beyond the advantage of reducing the disposal of and reutilizing material still fit for human consumption, an increase in potential health benefits has been observed *in vitro*. More *in vivo* studies are suggested to validate these findings. An example of nutritional benefit was offered in the study of Xie et al. in which wheat bran was fermented by the association of *Propionibacterium freudenreichii* and *Lactobacillus brevis*, obtaining significant amounts of vitamin B12 while maintaining the safety of the food substrate. These results encourage further exploration of microbial biosynthesis of bioactive compounds to fortify food ingredients of plant origin.

Fermentation of maize bran and germ with selected lactic acid bacteria starters previously isolated from different grains enhanced the quality of the raw material by decreasing the phytate content and the lipase activity. At the same time, free amino acids, peptide concentration and the antioxidant activity of fermented maize bran and germ were enhanced. The use of the pre-fermented by-products as ingredients for breadmaking extended the positive attributes to the bread (Pontonio et al.). Aside from common cereal grains, the increase of the nutritional and functional qualities of pseudocereals via lactic acid bacteria fermentation was reviewed by Rollán et al.. The importance of these crops lies in their resilience and adaptability to harsh climatic conditions, providing an important resource for crop diversification and sustainable agriculture. Among the advantages of fermentation, the decrease of antinutritional factors like phytic acid and the increase in phenolic compounds and B vitamins have been observed.

The reduction of food losses requires an integrated approach to be implemented at different steps of the food chain. Sarwar et al. studied the antagonistic ability of *Streptomyces violaceousniger* against the pathogens responsible for the potato common scab. The selected strain was effective in controlling the disease thanks to the biosynthesis of the antimicrobial compound azalomycin, while simultaneously enhancing the crop yield.

Algae and microalgae are emerging as valuable biomass for different applications, including food and feed. To increase their sustainable use, algal biorefinery processes and purification of bioactive compounds should become more efficient, reducing the environmental impact and side-stream formation. In the study of Chen et al., the extraction of oligosaccharides from the alga *Gracilaria lemaneiformis* was implemented by utilizing the agarolytic activity of *Flammeovirga* sp., a bacterial species inhabiting deep sea water. The resulting environmentally friendly fermentation process allowed the retention of to retain the algal beneficial compounds, reducing by-product formation. Patnaik et al. proposed a novel biorefinery approach to valorize the whole biomass of the microalga *Scenedesmus obliquus*, achieving the conversion of 70% of the microalgal biomass into an industrially relevant product. Furthermore, the positive impact of microalgal protein as feed supplement for fresh-water fish was assessed.

In the feed industry, finding new solutions to enhance the nutrient uptake from plant-based material could contribute to achieving more sustainable, completely plant-based feed, valorizing side-streams, and local resources. Dittoe et al. focused on the search for pectinases to degrade the plant cell wall. Several pectin-related enzymes were found in the genome of *Dickeya dadantii* together with a significant number of pectin degradation-related pathways. This strain could be used as a commercially viable enzyme producer.

Research on microbiome and its applications in the agricultural system has intensified in recent years due to the continuous advancement of genomic information. In the study of Dong et al., the effects of freeze–thaw events on microbial community dynamics and the fermentation quality of red clover silage were investigated. The freeze–thaw events increased the abundance of specific microbial groups at different stages of ensiling, suggesting how to control the quality of silages when forages are subjected to freezing damages. In the study of Wang et al., the capacity of a consortium of different bacterial genera isolated from wheat field in detoxifying deoxynivalenol was defined. The findings highlighted the potential of microbial biotransformation in improving feed and food quality and safety, contributing to a more resilient food chain. Finally, the microbiome along the soil-plant-animal continuum within the pastoral production system was reviewed by Attwood et al.. Understanding the microbial diversity within these environments can be used to improve agricultural processes, helping us to face the upcoming climate change and food security challenges.

The above studies represent an overview on the role of microorganisms as means to design novel industrial applications encompassing the whole food production chain. Microbial biotechnologies focusing on the reduction of food waste and loss and the improvement of process efficiency have the potential to deliver important innovations in the agro-food industry. In the future, we envision that better understanding of the microbiome will lead to a more holistic interpretation of ecosystem functionality, creating a more sustainable agricultural system.

## Author Contributions

All authors listed have made a substantial, direct and intellectual contribution to the work, and approved it for publication.

## Conflict of Interest

The authors declare that the research was conducted in the absence of any commercial or financial relationships that could be construed as a potential conflict of interest.

## Footnotes

^1^https://eatforum.org/eat-lancet-commission/

